# Revisiting the quality criteria for qualitative research

**DOI:** 10.1177/14550725251358087

**Published:** 2025-07-22

**Authors:** Matilda Hellman

**Affiliations:** Department of Sociology, 225313 Uppsala University, Uppsala, Sweden; 154260 University of Helsinki Faculty of Social Sciences, Helsinki, Finland

Four years have passed since we formulated a position on how we assess qualitative submissions to our journal (Hellman, 2021). That text included tables with checklists and practical advice. In this editorial I revisit this topic, pointing out some of the most common issues in current qualitative manuscript submissions.

First, I outline some general points about qualitative research, of which some will also come up in the conversation in a forthcoming podcast episode of *Publishing Addiction Science* (shows.acast.com/publishing-addiction-science). *Publishing Addiction Science* is a podcast produced by the Society for the Study of Addiction (SSA) and the International Society of Addiction Journal Editors (ISAJE), featuring interviews and conversations with people involved in academic publishing in the field of addiction.

Second, we have compiled a table with evaluation criteria (Table 1). These are partly structured modelling a recently published general evaluation framework of our sister journal, the Finnish *Yhteiskuntapolitiikka* (yplehti.fi, 2025). Our table outlines what characterizes a well-crafted qualitative article, as well as typical shortcomings in manuscripts that fall short. Each row addresses one of seven criteria, accompanied by practical explanations, common problems and concrete advice. The criteria cover the following areas: (1) Formulation of the research problem (RP) and the intended contribution of the study; (2) Familiarity with and presentation of existing research and theoretical discussion; (3) Quality of the data and clarity in describing the study's design and proceedings; (4) Quality of the results section and analytical depth; (5) Quality of writing and overall presentation; (6) Proportionality of the manuscript's length to its informational value, and internal coherence between theory, data, and results; and (7) Relevance of the theme to the journal's scope

**Table 1.** Overview of criteria, common mistakes and some recommendations regarding qualitative research contributions.

Criterion	What it means in practice	Typical problems to be avoided	Some tips and ideas for better fluency and more interesting text
1. Formulation of the research problem (RP) and the intended contribution of the study	The research problem (RP): is interestingly and clearly articulated in the beginning and the author sticks to it throughout and revisits it (at the very minimum in end discussion) paves the way for the reader to become engaged and wanting to know more involves a conversation of what is known and unknown, for example, through identifying gaps in prior research or academic discussions	The rationale of the RP is presented as in a quantitative article, purely based on mechanic accounts or empirical gaps Example 1: “*Problems X are widespread (research reference), and Y are an especially vulnerable group (references to research). This is why it is important to study Y's mental health*” The writer does not acknowledge a relative or dynamic reality but presents RP as existing in a stable form, independently of how scholars approach it. Example 2: “*Problem X is increasing* [an independent stable fact] *and this is why we need to know more*” Too much room given to too many circumstances so that the introduction is sprouting in many directions: Example 3: *The impact of COVID-19 on Problem X … Structural inequalities and poverty among … Group Y … cultural stigma … youth trends … access to help … the role of social media … neuroscientific explanations of depression … a critique of DSM-5 … gaps in qualitative methodology, etc.* [instead, these can be shortly enumerated as a reminder of relevance and complex reality surrounding the study focus]	The RP and the impetus for the study (“what's at stake”) should be justified and substantiated convincingly referring to general / philosophical / theoretical / everyday / – / etc. thought-provoking aspects of a phenomenon / life / the world / – / etc. Aim for a sense of “this is intriguing, this is truly important, I never thought about it in this way”. Align RP with qualitative design from the outset and accentuate (verb choices, angles of presentation) all aspects which speak to and require a choice of qualitative method and give less attention to the rest: *INSTEAD OF:* “*This study investigates how individuals Y experience workplace interventions after actions Z related to X violations*”*. *[experiences can be investigated also in other ways]* INSTEAD:* “*This study explores how individuals Y make sense of and navigate the kind of workplace interventions that follow X violations. This is captured in accounts of their lived experiences and subjective meaning-making of* …”*.* What we are striving at: When the reader arrives at the methods section they are already convinced of and have identified a need for a certain design choice. Use your imagination and think through / accentuate the principle question at stake: “*This pertains to a larger principle moral question of how we justify suffering among group Y considering that the local government has articulated ambitions to prioritize … How decision-makers and policy documents frame Problems X and ideas about the lifeworlds of Y shape the everyday well-being of Z…*” Stick to the story and leave out unnecessary details Keep RP specific enough to guide data collection, but wide and principle enough to feel urgent and interesting
2. Familiarity with and presentation of existing research and theoretical discussion	The author demonstrates that they understand the different empirical and theoretical discussions of which the RP and study is part, and is, for example, able to name them and describe their essence A well-written and thoughtful literature review makes it easy to grasp previous and current research conversations and dilemmas that the manuscript helps shed light on and solve. The existing research and theoretical backdrop are presented in informing, coherent, relevant and correct storylines.	It remains unclear which research discussion the manuscript engages with. Even if many current discussions and topical events can justify and scaffold the study's scope, the writer needs to decide about which ‘mix of circumstances’ to emphasize as most important and the main motivation that drives the narrative of the manuscript and guides the fundamental argumentation. Check, that this backdrop story is in line with what is added through empirical results and emphasized in the end discussion. Mechanic literature review of a massive amount of quantitative studies as if it was all equally relevant. The manuscript states that the phenomenon in question hasn’t been studied with similar data and methods before but does not justify why the chosen data or theory are suitable for solving or furthering insight on the research problem.	Name and describe the scholarly discussions as if they entail an interesting story on their own (but, still keep it relevant and stringent): *The sociological discussions in this area of research have especially concerned A,B and C. These traits rose in the 1980s with the advent of the linguistic turn.* In 1984, a *now famous study by sociologist W, pointed out that /* – */. In what followed some case studies have shown a more complex and nuanced picture of the situation /* – */. Sociologist seem to have been reluctant to shed light on … / – /. This might be the result of…* No need to mention all studies that you found, exemplify with and single out a selection that is relevant for the study and brings up interesting points and drives the structural story of the report: *Scholars have repeatedly argued that group Y are more sensitive due to the characteristics of X (see e.g., Kettunen 1995; Hellman 2009), but, in 2015, Lindeman pointed out that this could be framed in a totally reverse sense, emphasizing …*
3. Quality of the data and clarity in describing the study's design and proceedings	The manuscript utilizes a rich (empirical) data set with a sample logic and collection technique. The data and methods are likely to be fruitful and they make sense in view of RP and overall contribution of study The data are described in a way that allows the reader to understand their nature and extent and evaluate the strength and relevance of the overall contribution The use of the chosen data and method is justified in relation to the RP	The empirical data seem suspiciously scarce and small and / or the author does not communicate what exactly the sample represents, considering the great questions that the study promises to dig into, and / or in view of the heterogeneity of the targeted population The author's choices of approaches and proceedings do not seem thought-through and they are unable to express what the data can and cannot capture. A suspicion arises with the reader that the data may have been collected in a way that does not ideally serve the RP. Too structured and inflexible interview methods may for example not spontaneously invite more un- / sub-conscious conceptualisation that draws on the cultural material / individual / identity forming meaning-making that is sought to be captured The description of the data is vague or incomprehensible It is not explained why the data collection and analytical scheme are suitable for addressing the research problem or as part of the research design In theory section: Avoid justifying decisions by referring to other persons views on the matter (“*According to Spaniel 2009 this is a good method …*”. Instead, focus on how you find that the theory serves this particular study and how it is integrated into your analysis in your own reasoning. [naturaly, this argument can be strengthened by pointing out that others have come to the same conclusion in similar cases]	Smaller samples can be justified if they produce an added value in terms of new meaning-making, as in e.g. a conversation or interaction (focus group or free conversation) or if the researcher masters in-depth micro-analytical tools. Three shorter focus groups conversations with four individuals in each group (total N = 12) can with the proper analytical skills be a richer material than identical structured questions in lengthy in-depth interviews with 12 individuals. Specify and describe the data's extent and nature so that the reader gets a sense of it, for example in numerical terms*.* “*The total transcribed material amounted to 172 pages of text in a Word document with standard 12-point font*” Descriptions of and reflections surrounding the nature of the textual material and the selection of the analyzed parts will strengthen the scientific quality and convince the reader: *For this study we have only selected the parts of the material in which participants conceptualize questions related to how A is entangled with B. Large portions of the conversations (up to 35 transcribed text pages in total) were left out of this sub study as they revolved around question C and D, which were judged not to contain essential meaning-making related to …*” When explaining your choices of data and methods, stick closely to justifications that clearly support the specific aims and purposes of your study. Avoid general or irrelevant reasoning True theoretical integration means showing how the theory informs your research design, interpretation, or findings – not just praising its general merits.
4. Quality of the results section and analytical depth	The structure of the analytical account is comprehensible and logical The analysis provides a solid foundation for discussion and conclusions and contributes significantly to solving the research problem and shedding light on new aspects of the research question The limitations and relativity of the design or data are disclosed or discussed in a way that convince the reader of the researchers’ professionalism and knowledge of what they are doing	A thematic analysis that is purely a descriptive mapping is just a material description and belongs in the material section. A raw thematization is merely a first mapping of the material and does not yet constitute a proper qualitative analysis Lack of “science-making”: The researcher takes as their task to inductively identify themes and these themes are presented as results. There are no explanations of what they mean and how they relate to larger principle questions in RP. Patterns are highlighted without linking them to what they mean. If you only want to identify patterns, sometimes quantitative tools are a better choice Presentation of analytical results are not in sync with theory, analytical tools and methods Data extracts seem ad hoc and are listed without interpreting their significance; the body text merely repeats the content	Explain why you are conducting a thematic analysis how you interpreted the importance of different parts of it (the meaning-making and conceptualizations related to theoretical framing and RP) and why it is relevant: “*The themes and conceptualizations surrounding Phenomenon Y stood out as the most important meaning-making dimension of the interviewed ABC discourse among individuals Y. This line of reasoning entailed reflections on a connection with X, while emphasizing especially A and B. These elements were often connected logically in the speech of the study participants, as demonstrated in the following quote:* ‘—'”. After the quote, explain what it means or brings about in the bigger picture and the overall storyline of your presentation. Exercise your analytical writing skills by reading and trying out methods of how to argue, present interpretations and how to mediate a sense of reliability and honesty
5. Quality of writing and overall presentation	The manuscript uses proper and well-articulated English language The text predominately follows the logical structure for social science articles (introduction, literature/theoretical framework, research design, results, discussion, conclusions), and deviations do not stick out or feel unmotivated to the reader The reference list and other technical matters adheres to the journal's guidelines	The language being sloppy is a bigger problem than being overly bureaucratic or containing excessive theoretical jargon. For editors and reviewers, it is easier to trust that an author can make abstract writing less abstract than to trust that they can elevate a plain and uninteresting text to a higher quality level The reasoning and presentation are not unique and original, but feels self-evident, boring and / or superficial The sentences and paragraphs do not follow each other logically, making the text come across as confused or disjointed The manuscript's structure does not follow a logical / the journal's format The introduction and conclusions do not logically correspond and do not feel as part of the same coherent story. The manuscript ultimately fails to answer the posed research question and leaves the reader perplexed about what the actual contribution is.	Practice writing skills and read / study texts that are well-written A text is never finished: it is just better or worse than its previous and forthcoming versions. Use language reviewers, computer-based translation and editing services or AI tools trying out other ways of saying things (in active voice instead of passive, for example) and rewrite sentences and paragraphs. But don’t forget to declare how AI-tools are used in language processing and writing Stephen King's topic sentence technique emphasizes clarity, flow, and anchoring each paragraph around a clear central idea (the “topic sentence”), followed by supporting detail: “*Group Y tend to experience X not as a fixed state, but as a dynamic interplay between connection, stimulation, and safety [=* *Topic sentence]. In the interviews, X was most often described through moments of C, B and A, / – / etc. But these emotional ties were tightly interwoven with expressed needs for D, F and G translating to an access to E and to a sought-for interaction with Q or other Ys / – / When these elements aligned, the interviewees expressed that they were* ‘*radiantly content*’ *or* ‘*completely themselves*’*. It was in the small, repeated rituals of daily life that they say X as materializing and becoming visible …*”
6. Proportionality of length to informational value and internal coherence between theory, data, and results	The manuscript includes essential and relevant references to previous and ongoing discussions, literature and empirical findings The analysis is presented interestingly, clearly and concisely and supplementary materials are used when needed Key concepts are defined, and their internal relationships, functions and roles in the study and manuscript are clear and make sense The research design is well grounded and motivated Everything is logical and coherent: the things promised in the beginning are addressed and revisited.	The parts of the manuscript do not form a coherent whole The manuscript contains many irrelevant sidetracks and discussions and concepts that are not related to the RP or the study's storyline, aims and objectives The discussion or conclusions include a lot of new information or new theoretical concepts that is not supported by earlier sections and make the reader confused The manuscript reports data analysis results like a technical report without integrating them into the overall narrative of the manuscript, or without operationalizing key concepts, deriving them instead directly from the data The manuscript does not provide a sense of the researcher being reflective about the choices they make and understand how to build up motives and justifications for proceedings	Take breaks from writing a manuscript or alternate between different authors and readers; you can get blind to your own work and not understand how a reader comprehends the story and the structure. Have peers or mentors read your draft focusing solely on coherence, and ask them to point out sections that feel disconnected Outline your manuscript early. Draft a detailed structure showing how each section connects logically to the next Use signposting and transitions. At the end and beginning of sections, briefly explain (to yourself) how they relate to each other. Check that all paragraphs are natural continuations of the previous one and a natural base for the following one Constantly revisit your research questions. Make sure each section relates to and addresses the core questions or objectives Create a “thread” or narrative arc or a line of concepts that are being introduced that runs through the entire manuscript — each part should contribute to developing the main argument Be ruthless with editing. After writing a draft, go paragraph by paragraph asking: “Does this directly support the research question or the study's purpose?”. Kill your darlings! Write a focused research question(s) or aim(s) and keep it visible (e.g., on a sticky note by your computer) Use a concept map to visually track which concepts relate to your research aims; discard anything outside this map Set limits on literature reviews or theoretical discussions. Only include those directly relevant to your research aims If unsure about a section, label it “to review later”. After finishing the draft, review all such sections and cut or rewrite as needed Check all discussion points against earlier results or literature sections. Every new claim must be traceable back to data or previously presented evidence or points made in the overall study objectives Avoid introducing new concepts or data in the end of manuscript or in the discussion section. If new information arises, consider adding it earlier in the manuscript. Use “summary tables” or bullet points to explicitly link findings to discussion points before writing the discussion. Draft a “reverse outline” of your discussion to check if all points are grounded in earlier content.
7. Relevance of the theme to the journal's scope	The research contributes to new, societally / social scientifically relevant and interesting insights or a significant finding in view of social scientific aspect(s) of phenomena related to substance use, gambling, addiction and policies and systems in place for preventing and taking care of problems related to these behaviours There is some aspect of the research that more or less pertains to a Nordic context or questions that have been of importance in a Nordic context (this is matter of interpretation and NAD is generally positive to contributions from outside the Nordics)	Repetition: Key findings have already been published elsewhere (domestically or internationally). The results or their relevance are not connected to a societal phenomena The study is not social scientific to its character but is for example a clinical, pharmacological, medical or psychological study.	Rework your text and edit it so that it fits the journals scope and tone. Be especially thorough with title and abstract – these are the visit card of your submitted text, don’t leave them to the last minute. Check out the previously published contributions and their scopes and styles When choosing journal, note whether the journal leans more toward empirical work, theoretical discussions, policy relevance, or methodological innovation. (NAD focuses for example on many of these) Emphasize the parts of your study that speak most directly to the journal's focus If unsure, you can send an inquiry to the editor who can help you assess fit before submitting. Mention your topic and ask if it aligns with the journal's current interests. The editor can explain how the editorial office usually reason around their scope

Reference citations in Table 1 are usage examples only.

## Qualitative addiction research

There are aspects of the world that simply resist being captured in neat numbers and statistical categories. When our research foci concern meaning, understanding or the ways things unfold in context – how people make sense of their experiences, how institutional praxis is permeated with normative thinking, how discourses are shaped – we turn to qualitative research. It's not because we oppose measurement, but because quantifying in every instance can flatten or obscure.

Qualitative research entails an awareness of how the researcher and their decisions are part of the study and affect the studys scope and results. To borrow a metaphor, as qualitative researchers, we are not only primarily interested in naming and categorizing the colours in a spectrum, but we are interested in how they might look different depending on who is doing the seeing and how the colours came to be named, what they mean, etc. Qualitative research requires us to be attentive to complexity and meaning and invites us to reflexivity: a recognition that the researcher is not a neutral observer, but always shapes the research through presence, questions and interpretation.

The boundary between qualitative and quantitative research is blurrier than many assume. The most reflective quantitative researchers – social epidemiologists, for instance – are constantly expressing deep awareness of what their models cannot capture and they engage in conversations about the limits of their tools. Conversely, qualitative researchers do recognize and look for patterns, frequencies, even descriptive counts, as long as these are not mistaken for explanation in themselves. Qualitative scholars can adopt the critical self-scrutiny that many quantitative researchers apply to methodological rigor and contribution. Quantitative researchers can enrich their theoretical imagination and narrative craft by engaging with qualitative work.

The most common quality flaw by the qualitative submissions that we receive at the journal is being too descriptive: repeating what seven persons said in interviews without analytical framing or theoretical depth might produce a compelling news article – but not a scholarly study. If you simply report that four participants found something “good” and three found it “less good”, one must ask: was an interview even necessary, or could a survey box have done the job?

Collaboration, skills training and mentorship matter in qualitative research: For early-career researchers, finding exemplary texts, reading widely in theory and methodology, and co-writing with senior scholars can be transformative. Above all, as qualitative scholars, we need to be writing, writing and then doing some more writing. Reading philosophical arguments and wrestling with interpretation sharpen the analytic edge. AI tools can support language review and even offer feedback on clarity of argument, but they cannot replicate the creative, context-sensitive thinking that defines strong qualitative inquiry.

Our recommendations come with a content warning: qualitative research is a craft – of words, ideas, and the skillful communication of meaningful insights about the world. It is challenging to guide and even harder to fully explain in detail: writing, practising research methods and analyzing melt together as one and the same craft.

## In this issue

A study by Luurila et al. (2025) describes workplace interventions for registered nurses with substance use disorder-related violations that have resulted in disciplinary actions. and Marionneau et al. (2025a) argue the benefits of a standardised data collection practices for gambling helplines and, in an overview report, it is asked whether there is a Nordic model for the regulation of alcohol and gambling (Marionneau et al., 2025b). In a cohort study, Nesse & Clausen (2025) unfold the treatment satisfaction among patients in opioid agonist treatment in Norway. Rúdólfsdóttir & Guðjohnsen (2025) have interviewed young people about their alcohol consumption.
